# Sub-pixel correlation length neutron imaging: Spatially resolved scattering information of microstructures on a macroscopic scale

**DOI:** 10.1038/srep44588

**Published:** 2017-03-17

**Authors:** Ralph P. Harti, Markus Strobl, Benedikt Betz, Konstantins Jefimovs, Matias Kagias, Christian Grünzweig

**Affiliations:** 1 Laboratory for Neutron Scattering and Imaging, Paul Scherrer Institut, 5232 Villigen, Switzerland; 2 University of Geneva, 1211 Geneva, Switzerland; 3 European Spallation Source E.R.I.C., 22100 Lund, Sweden; 4 Niels Bohr Institute, Copenhagen University, 2100 Copenhagen, Denmark; 5 Swiss Light Source, Paul Scherrer Institut, 5232 Villigen, Switzerland; 6 Institute for Biomedical Engineering, ETH Zurich, 8092 Zurich, Switzerland

## Abstract

Neutron imaging and scattering give data of significantly different nature and traditional methods leave a gap of accessible structure sizes at around 10 micrometers. Only in recent years overlap in the probed size ranges could be achieved by independent application of high resolution scattering and imaging methods, however without providing full structural information when microstructures vary on a macroscopic scale. In this study we show how quantitative neutron dark-field imaging with a novel experimental approach provides both sub-pixel resolution with respect to microscopic correlation lengths and imaging of macroscopic variations of the microstructure. Thus it provides combined information on multiple length scales. A dispersion of micrometer sized polystyrene colloids was chosen as a model system to study gravity induced crystallisation of microspheres on a macro scale, including the identification of ordered as well as unordered phases. Our results pave the way to study heterogeneous systems locally in a previously impossible manner.

Conventional neutron scattering techniques such as Small-Angle Neutron Scattering (SANS) give information about sizes from Ångstroms to few hundreds of nanometres^1^. The global nature of the data limits the application to samples that are either homogeneous or heterogeneous on a scale much smaller than the illuminated part of the sample. On the other side neutron imaging reaches down to about 20 μm with field of views in the centimetre range^2^,^3^. The gap between imaging and scattering is accessible by advanced scattering techniques such as Ultra Small Angle Neutron Scattering^4^ or Spin Echo SANS (SESANS)^5^. These techniques approach the gap in the reciprocal space domain with the drawback of not providing spatial resolution (macroscopic) of individual structures (microscopic). First attempts to combine imaging and scattering have been made with the development of Spin-Echo Modulated SANS (SEMSANS)^6^ which covers the same microscopic size range as SANS with low spatial resolution of only 1 mm. Great effort is currently put into resolving smaller structure sizes directly by pushing the imaging resolution limit, in a small field of view of 3 mm × 3 mm, down to 5 micrometres with the development of neutron microscopes^7^,^8^,^9^.

Besides traditional neutron imaging the dark-field image (DFI) in neutron grating interferometry (nGI) is known to contain information on structure sizes smaller than the resolution limit. The highest sensitivity for DFI, indicated by the probed autocorrelation length, in conventional nGI setups has experimentally been determined to be around 2 μm^10^. Recently a general theoretical solution for the image formation in DFIs was presented^11^ which paved the way for the extraction of correlation functions that describe the full microstructure of the sample. The feasability has been demonstrated for neutrons by Strobl *et al*.^12^ and for X-rays by Prade *et al*.^13^. However these studies are proof of principle analysis performed with reference samples and merely illustrate the possibility of extracting an averaged scattering function from a significantly large part of the field of view without in particular exploiting the spatial resolution capabilities, thus losing the imaging character of the technique. No successful utilization of the spatial resolution has yet been presented.

In this article we introduce an advanced quantitative DFI approach that is based on a modified nGI enabling 2D sub-pixel resolution of autocorrelation lengths (*ξ*) on an extended microscopic range while maintaining the full spatial resolution capabilities in real space. A variation in *ξ* makes it possible to extract the distance-dependence of the statistical ensemble average^14^ and hence the determination of the autocorrelation function of the system. This allows the investigation of individual, local variations of microstructural features on a macroscopic scale down to 90 μm. We call this technique sub-pixel correlation length imaging (*ξDFI*). By the use of *ξDFI* we do not only overcome the limitations of scattering (no spatial resolution) and imaging (no microstrucutral information) techniques, but also those of earlier attempts such as wavelength dispersive DFI^12^ and SEMSANS, which for long correlation lengths quickly loose both the scattering sensitivity and sufficient spatial resolution. In particular we record images with a monochromatic beam in which each pixel contains a scattering function that carries structural information on sample features smaller than the imaging resolution itself and we hence decouple, in contrast to earlier approaches, the resolvable feature size in both domains from a wavelength variation^6^,^12^. Because each pixel carries information about the scattering structure we can extract the spatial distribution of the scattering signal. Thus we observe a local change in microscopic structure over the whole field of view of the imaging setup (in our case up to 64 mm × 64 mm)^2^,^15^ without compromising spatial image resolution. With that the characterization of heterogeneous samples, non-model, non-lab optimized systems becomes possible in a previously inaccessible manner.

To demonstrate the potential of *ξDFI* we analysed a system of polystyrene microspheres that is often used as a model system for the investigation of crystallisation processes^16^,^17^. In particular we study gravity induced sedimentation that leads to a system with unordered, as well as ordered phases and even shows phenomena such as an extended depletion zone. Similar processes are often used for fundamental studies on crystallisation and more applied work in the field of optical band-gap crystals and protein crystallisation^18^.

We used neutrons instead of X-rays for their advantage of tuning both the macroscopic mass density and the scattering length density of the solvent at the same time, without changing the particle density by simply adjusting the solvent of water (*H*_2_*O*) and heavy water (*D*_2_*O*). Thus we could introduce sedimentation and then follow the local changes in microsphere density and structure. The formation of ordered structures from unordered microspheres in solution is an example of the crystallisation of micrometre sized particles and is a direct consequence of the favorable energetic state of the crystalline structure over the unordered arrangement.

In the following we describe the modified nGI setup that we developed for the realisation of *ξDFI* measurements. Afterwards we will introduce the fundamentals of an experimental approach that allows the full characterisation of the sample microstructure, followed by the experimental study of the sedimentation of polystyrene microspheres that utilises the additional capabilities of *ξDFI*.

## Modified neutron grating interferometer and sample preparation

**Figure 1:**
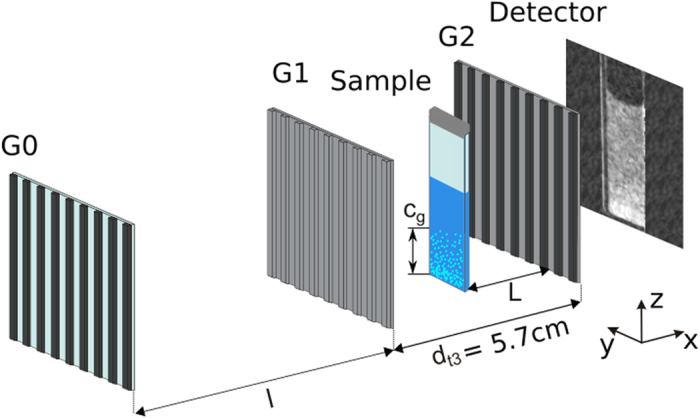
Sketch of the modified Neutron Grating Interferometer setup for the third talbot distance. The setup uses a *G*_*0*_ grating as source grating, a phase grating (*G*_1_) at distance l = 5.23 m and an analyser grating *G*_2_ at distance *d*_*t*3_ from *G*_1_. With a *d*_*t*3_ that resembles the third talbot distance (5.7 cm) we could place the sample between *G*_1_ and *G*_2_ and vary the distance *L* between sample and *G*_2_. *c*_*g*_ indicates the concentration gradient in solution of sedimented particles.

Neutron grating interferometry (nGI)^15^ is an imaging technique which simultaneously provides the conventional attenuation based transmission image (TI) and the scattering based dark-field image (DFI)^19^,^20^,^21^. A schematic of a modified nGI setup is shown in Fig. 1, including gratings as well as the sample, positioned between G1 and G2, and the detector behind G2.

nGI images are generated by creating an array of line sources with an absorption grating (G0) and then introducing a phase shift by means of a phase grating (G1). This leads to an interference pattern that is too small to be detected with current neutron imaging detectors. An analyzer grating (G2) with the period of the generated interference pattern is then used to detect the intensity variations of the interference pattern with a scanning procedure.

Dark-field imaging with nGI gained great interest in recent years with the study of magnetic structures^22^,^23^,^24^,^25^ as well as experiments in which the contrast origin lies within the microstructure of the sample^10^,^19^,^26^,^27^. All previous nGI experiments used setups in the first Talbot order, which has limitations when it comes to the selective positioning of the sample in beam direction. As a consequence previous DFI studies were performed at fixed sample to G2 distances (L). Using the setup, that is illustrated in Fig. 1, the sample was positioned between G1 and G2 which was made possible by building a setup in the third Talbot distance and hence increasing the distance between G1 and G2 from dt1 = 19 mm to dt3 = 57 mm. The third Talbot distance setup presented here also allows the recording of images at a single L, but extends the capabilities of the setup and makes it possible to vary the sample to G2 distance. A variation in L can be important to tune the sensitivity of the setup^28^ and can be used for the quantification of the dark-field signal^11^,^29^.

The *ξDFI* technique in combination with the modified nGI setup was applied to a solution of 3 μm polystyrene microspheres in which gravity driven sedimentation was induced. The microspheres are NIST traceable polystyrene microspheres purchased from Magsphere and are typically used for calibration and standardistation. They are delivered in an aqueous solution with 0.1% of sodium azide as preservative. In order to verify the diameter reported by the manufacturer we prepared a sample in which the density of solution and microspheres are matched at a particle concentration of 8.55% and repeated measurements of the type proposed in refs 12 and 13. The results and model description can be found in Supplement 1. Polystyrene microspheres have a macroscopic density of 1.05 g cm^−3^ and a scattering length density (SLD) of 1.412 · 10^−6^ Å^−2^ 30. The sample container was a 5 mm thick quartz glass cuvette and the sample for the sedimentation experiment had an initial particle concentration of 11.9%.

In order to achieve good contrast and to create a solvent with a density only slightly smaller than that of the microspheres, we mixed 21% *D*_2_*O* (SLD = 6.393 · 10^−6^ Å^−2^) and 79% *H*_2_*O* (SLD = −0.561 · 10^−6^ Å^−2^) with a combined scattering length density of 0.899 · 10^−6^ Å^−2^ 30 and a mass density of 1.02 g cm^−3^. Thus the time scale for the colloids to sediment was in the order of days and due to the small mismatch in mass density the continuing sedimentation process happened slowly. The sample was contained in a closed system with a sealed cuvette and constant temperature at 25 °C and an ambient humidity of 18%. Hence, our measurements could be performed on a quasi-static system.

## Single sample-to-G2 distance DFI measurements

**Figure 2:**
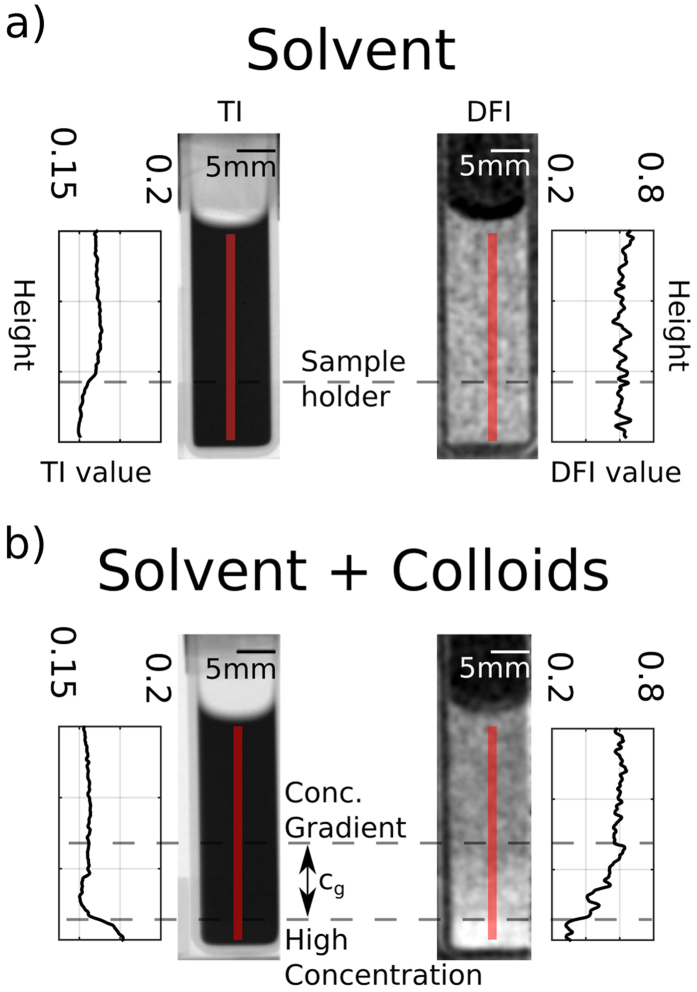
Single transmission image (TI) and dark-field image (DFI) at fixed L = 0.9 cm of pure solvent and solvent with sedimented 3 μm polystyrene spheres. (**a**) TI and DFI of the solvent inside a quartz glass cuvette with corresponding vertical line scan following the red line. (**b**) TI and DFI of solvent with sedimented colloids and corresponding line scan. The increase/decrease on the bottom of the TI/DFI of (**b**) indicates a concentration gradient of microspheres. The concentration gradient *c*_*g*_ within the sample is indicated. Below *c*_*g*_ an area of high concentration is shown.

At first a qualitative measurement of the sedimented solution of microspheres was conducted with a single sample-to-G2 distance L. Figure 2 shows the results of the nGI measurements with the minimum L = 0.9 cm. The limit for the smallest L is set by the lateral dimensions of the sample holder. Figure 2a presents both the TI and the DFI of the solvent without microspheres. The corresponding line profile along the red line through the TI image of the solvent exhibits no significant changes over the sample height with a constant value of around 0.17. Only the aluminium sample holder (dashed line) contributes to a small signal decrease in the order of 5%. In case of the DFI of the solvent no signal variation is visible (DFI value of 0.6). This is due to the sample holder being made of aluminium, which does not display any significant ultra small angle scattering features.

The vertical line profiles of the images with 3 µm spheres sedimented at the bottom of the cuvette, Fig. 2b, show more pronounced features. Again the sample holder is visible in the TI, but this time there is also a significant increase in the signal at the bottom of the cuvette, where the microspheres sedimented. This is explained by a high concentration of polystyrene microspheres on the bottom of the cuvette. The attenuation coefficient of polystyrene (3.937 cm^−1^ @ 4.1 Å) is lower than that of the solution (4.290 cm^−1^ @ 4.1 Å)^30^.

Because the DFI signal originates from scattering within the sample and is concentration dependent^10^ we can see a decay of the signal beginning from the dashed line indicating the area with a concentration gradient (Fig. 2b). Above that the value stays constant and is comparable to the solution without colloids, indicating that there are no colloids present above the concentration gradient. In contrast to the TI the DFI shows a more pronounced change in signal. The DFI drops by 40%, whereas the TI only changes by 3%. The higher dynamic range of the DFI increases the visible contrast significantly. The decay of the DFI value indicates an increase in concentration from top to bottom in *c*_*g*_. Below that region the signal is constant, indicating no further change in concentration.

However single sample-to-G2 distance DFI measurements can only provide the location of increased scattering but no detailed information on the scattering structure itself. In the following we present how an extension of the existing image acquisition procedure plus a modified nGI setup for the third Talbot distance allows us to extract the real-space correlation function of the system by scanning the sample to G2 distance L. A variation in L is important to tune the sensitivity of the setup^28^ and can be used for the quantification of the dark-field signal^11^,^29^.

## ***ξDFI***: Data acquisition and data analysis

Donath *et al*.^28^ pointed out that the distance between sample and gratings strongly influences the retrieved signal. Later it has been shown by Strobl^11^ that the distance L is one of the parameters defining the correlation length probed by a specific measurement and that hence a scan of this parameter enables systematic quantitative scattering studies.

Traditional nGIs have a very long distance between G0 and G1 (5–10 m) with the sample being positioned between them. We modified the setup such, that it can be used at the third Talbot order, leading to a distance of ~5.7 cm between G1 and G2. The increased distance between G1 and G2 allows us to position the sample in between them and vary the sample to G2 distance L. As pointed out in ref. 28 the range of sensitivity change, by varying the sample-to-G1 distance between G0 and G1, has the same effect as varying the sample-to-G2 distance between G1 and G2, thus making the same range of sensitivity accesible over a few cm instead of meters in previous instrument designs. Furthermore large sample-to-G2 distances would increase geometrical blurring and thus spatial resolution would have to be sacrificed in case of varying the sensitivity with a sample positioned between G0 and G1.

In order to realise the new third Talbot distance design a set of gratings was produced that fulfill the requirements defined in ref. 15. With our third Talbot distance setup we can change L without affecting the spatial resolution significantly and sample position changes in the range of cm allow us to characterise micrometer sized structures.

For the characterisation of microstructures, which are below the pixel resolution of the imaging setup, individual images must be recorded at a variety of different sample to G2 distances *L*. The relation between the probed autocorrelation length *ξ* and L is described in detail in ref. 11 and is given by:


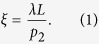


Hence scanning of *L* directly implies a scan of the autocorrelation length *ξ* at which the image is recorded for constant wavelength *λ* and period *p*_2_ of G2.

The change in DFI signal in each pixel (*n,m*) as a function of *ξ* can be expressed by ref. 11


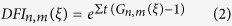


where ∑ is the macroscopic scattering cross-section and *t* the sample thickness. *G*_*n,m*_(*ξ*) is the one-dimensional projection of the autocorrelation function of the scattering structure in each pixel, calculated via the Abel transform of the autocorrelation function^31^. *G*_*n,m*_(*ξ*) can be interpreted as the real-space equivalent of the reciprocal scattering function *I(q*), which is measured, over the illuminated sample volume, in reciprocal scattering techniques such as small-angle scattering (SANS). In contrast to the global SANS technique the *ξDFI* approach provides the autocorrelation function of scattering structures pixel-wise and thus enables small-angle and in particular ultra-small angle scattering studies with additional macroscopic image resolution.

In the colloidal solution, that was studied during the experiments presented here, we observe a concentration gradient as presented before in (Fig. 2b). The variation of concentration directly influences ∑, but also *G*_*n,m*_(*ξ*)^11^,^32^, and according to Eq. 2 impacts the DFI, dependent on the probed value of *ξ* (Eq. 1). The concentration dependent DFI can be theoretically described by using the well known hard sphere model^33^. In scattering theory the scattered intensity *I(q*) for monodisperse isolated spheres is given by


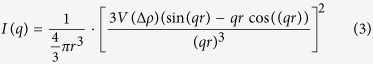


with *q* being the reciprocal scattering vector, *V* the volume of the scatterer, *r* the radius of the spheres and Δ*ρ* the scattering length density contrast, i.e. *I(q*) provides the form factor. In order to describe the behaviour of *I(q*) for higher concentrations the structure factor needs to be included either using the Perkus-Yevick closure^34^ for amourphous/liquid-like phases or a crystalline model to predict the impact of inter particle correlations for monodisperse spheres. The sphere model in combination with the structure factor *I(q*) can be translated to G(*ξ*) by a hankel transformation^31^:


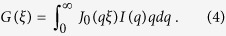


Here *J*_0_ is a bessel function of zeroth order and first kind. Thus we can model the change in greyscale of dark-field images as a function of *ξ* and correspondingly of L (Eq. 1), for monodisperse spheres of any concentration by inserting Eq. 4 in Eq. 2. Two examples that illustrate the conversion of reciprocal to real space can be found in Fig. S2 in Supplement 2.

**Figure 3:**
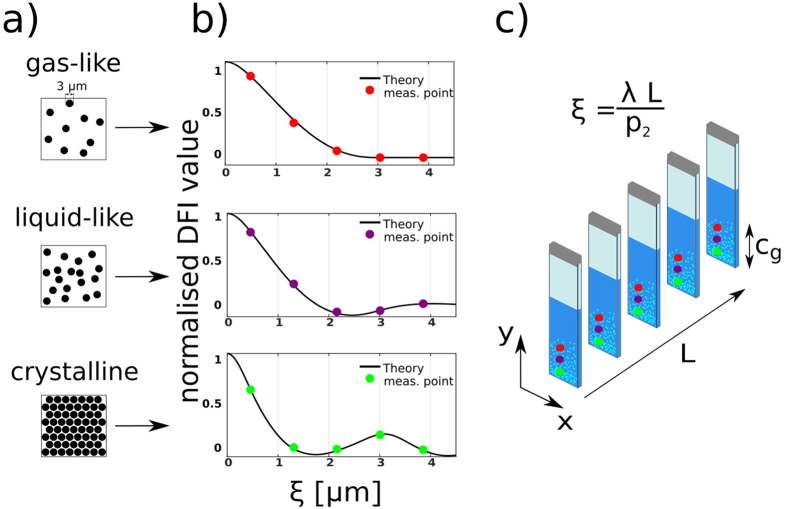
Schematic representation of the model description of increased concentrations of microspheres as well as the data analysis of dark-field image acquisition matrix with varying autocorrelation length *ξ*. (**a**) Real space representation of the model system for three different phases (gas-like, liquid-like, crystalline). (**b**) Theoretical model description of the DFI values as a function of *ξ*, with discrete measurement values indicated as points. (**c**) Illustration of the measurement procedure where the whole sample is scanned at various L from 0.9 cm to 4.3 cm.

The model description for increased concentrations of spheres in solution and its impact on *DFI*_*n,m*_(*ξ*) is illustrated in Fig. 3. Figure 3a presents schematic illustrations of three different phases: gas-like (isolated particles, no structure factor), liquid-like (particles with a concentration of 20%, Percus-Yevick structure factor) and crystalline (face-centerd-cubic structure as described in the methods section). As pointed out in ref. 35 the hard sphere model reaches its limit when it comes to the study of solutions with high concentrations as electrostatic screening leads to the necessity of using an effective particle diameter in the model descriptions. In order to incorporate such effects we used an effective radius to describe the peak positions of *G(ξ*) in case of the crystalline phase. In Fig. 3b the calculated *DFI*_*n,m*_(ξ) (black, solid lines) for the different phases are presented, according to Eq. 4. The differently coloured points (red, purple, green) illustrate the measurement procedure in which individual points in the real-space correlation function are probed by varying L, as illustrated in Fig. 3c. This results in a three dimensional image acquisition matrix with the 2D images and L as the third dimension. The red dot shows an area in the sample representing the gas-like, the purple dot the liquid-like and the green dot the crystalline phase.

**Figure 4:**
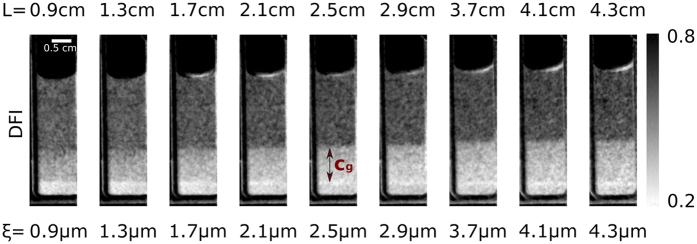
Dark-field images of a sedimented solution of 3 μm polystyrene spheres recorded at varying distance L and corresponding autocorrelation length *ξ*. *c*_*g*_ indicates a concentration gradient of microspheres. Above *c*_*g*_ no contribution of particles to the DFI signal can be observed, indicating the presence of the pure solvent.

In order to study the colloidal suspension presented here we recorded data as illustrated in Fig. 3. The DFI behaviour with varying *L* can be seen in Fig. 4, where a selection of 9 DFIs, out of a total of 18, is presented. The image acquisition matrix was processed with a 3 × 3 × 3 pixel 3D median filter, to smoothen the data. This type of filter is justified as its impact on the resolution is still significantly smaller than the area later used to extract the local scattering functions from the images. From left to right (in Fig. 4) the distance L between the sample and G2 is increased from 0.9 cm to 4.3 cm. This directly translates to a change in *ξ* from 0.9 μm to 4.3 μm according to Eq. 1. The cuvette does not contain microspheres above *c*_*g*_ and does not give any signal related to the them. In *c*_*g*_, however, the presence of microspheres increases the signal significantly. The most severe change, with varying L, is visible in the first few images of the scan. At large *ξ*-values, especially in the upper part of the concentration gradient *c*_*g*_, a saturation of the signal can be observed. This *ξ*-scan allows us to extract scattering functions, that contain quantitative information, within selected areas of the image, as shown in the following.

## Quantitative sub-pixel correlation length imaging of gravity induced crystallisation

As a consequence of the scattering related nature of the *ξ*-scan we can observe an increase in concentration as well as the crystallisation process itself. Structural changes, which are a consequence of crystallisation in the sample, severely impact the shape of the extracted real-space correlation function. Crystallisation and the corresponding ordering leads to a decreased inter particle distance as compared to isolated spheres, which can be detected and has significant impact on the recorded real-space scattering functions.

**Figure 5:**
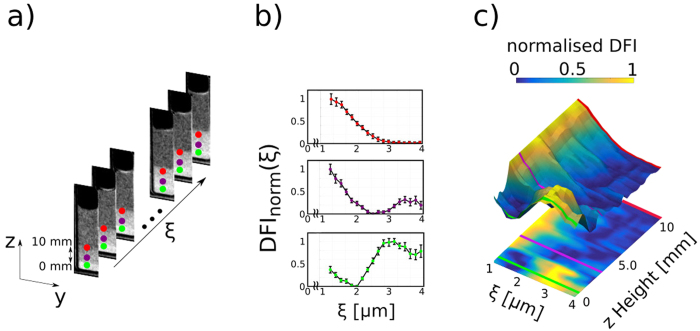
Extracting spatially resolved real-space correlation functions of a sedimented solution of microspheres including the identification of three phases of colloidal structure. (**a**) Data acquisition procedure. (**b**) Three extracted real-space correlation functions representing the gas-like (red), liquid-like (purple) and crystalline (green) phase. (**c**) Height profile of real-space correlation functions from 0 mm to 10 mm in z direction and the 2D projection of the colour-coded data.

Figure 5a shows the measurements corresponding to the theoretical considerations presented in Fig. 3. In Fig. 5b three different *ξ*-scans are extracted at different positions in the sample and are presented to visualise three different phases within the sample. The extracted DFI values are scaled according to


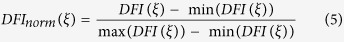


in order to visualise the impact of the structure factor. The impact of concentration changes is hence seen purely for G(*ξ*) and the effects on the macroscopic scattering cross-section ∑ can be ignored, because it is removed by the scaling. Figure 5c shows the colour-coded height profile of the real-space correlation function within *c*_*g*_, which will later be used to study the transition between the phases. It is extracted between height 0 and 10 mm and plotted as a continuous evolution of the real-space correlation function over the height of the sample. To simplify the visual representation the colour code is projected into a 2D plot. Furthermore the individual phases of Figs 3b and 5b are indicated by lines with the corresponding colours.

The top plot (red dots), in Fig. 5b, resembles a state of isolated particles with low concentration. This can be seen from the saturation of the *ξ*-scan signal at the diameter of the spheres in solution (3 µm)^11^,^12^,^32^. The fact, that the signal is stable and does not increase anymore for higher *ξ*-values proves that the concentration is so low, that no inter particle correlation is probed. The middle plot (purple dots) shows an increased concentration, to such extend that inter particle correlations are measured. This can be seen by the increase in DFI signal for larger *ξ*-values. Such behaviour can be modeled by increasing the concentration in the SAS model for hard-spheres (middle plot in Fig. 3b).

The bottom plot (green dots) shows another characteristic area within the region of sedimented colloidal particles. It can directly be compared to the green plot in Fig. 3. The peak does not only increase relative to the rest of the curve and in particular with respect to the previous case, but also shifts position towards a *ξ*-value of 3 μm. Such behaviour indicates a different phase and can be considered a densely packed crystalline structure, because the peak at 3 μm suggests an increased correlation that can be explained by closely neighbouring spheres, which can only be achieved in a hexagonal or face-centered close packing structure^32^. Further support for the crystallisation to happen is the relatively low Peclet number of 3.6 · 10^−4^ for the system. The Peclet number was calculated by 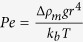 with mass density difference Δ*ρ*_*m*_, gravitational acceleration *g*, colloidal radius *r*, Boltzmann constant *k*_*b*_ and Temperature *K* as 298 K. A low Peclet number suggests a big influence of brownian motion, which supports crystallisation^36^. The possibility for such interpretation was previously inaccessible with single sample-to-G2 distance measurements, as these could only indicate the amount of scattering present in the sample but not indicate a phase change.

## Detailed analysis of the spatial evolution of phases

**Figure 6:**
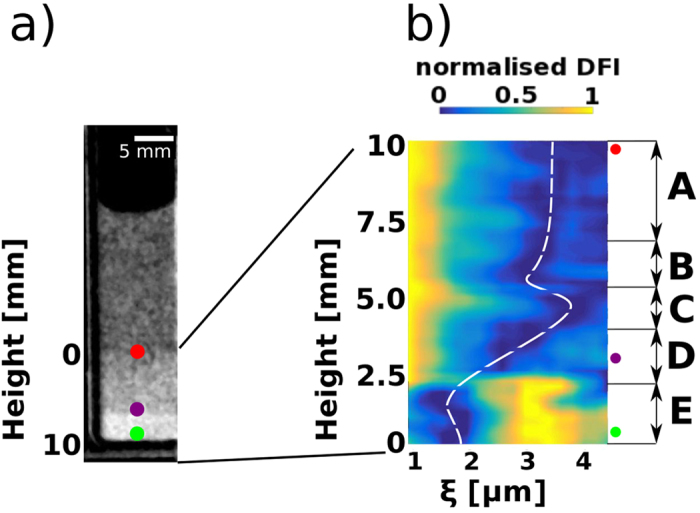
Evolution of phases in a sedimented microsphere solution visualised by the height dependent variation of the real-space correlation function. (**a**) Individual DFI image of the sample at L = 0.9 cm. The points indicate the areas of extracted real-space correlation functions in Fig. 5b. (**b**) Height profile of the real-space correlation function within the sample. Five areas of sedimentation can be identified (A,B,C,D,E). The dashed white line indicates the minimum at each height and is a measure for the concentration gradient.

The full potential of the 2D spatially resolved *ξDFI* data can be used by extending the data analysis from looking at the three different phases that are present in the sample to the transition between them. Figure 6a shows a single DFI taken at L = 0.9 cm with the three positions, discussed in the previous section, marked correspondingly with a red, purple and green dot. In Fig. 6b the colour coded projection of the 3D plot in Fig. 5c onto a two dimensional plane is provided. The dashed white line indicates the minimum of the normalized measured correlation function for every height of the sample. The data presented in Fig. 6b are a running average of correlation functions from top to bottom of *c*_*g*_ with a vertical step width of around 120 μm and a subset height of 750 μm. This way the real space correlation function, as measured by the DFI signal (Eq. 2), can be represented and examined with respect to its dependence on the height of the sample. Thus we can move from a mere observation of three phases within the sample to a spatial evolution of phases.

The detailed analysis of the evolution of phases within the sample indicates the existence of five different areas (A-E) of heterogenous crystallisation, as shown in Fig. 6b. Area A is characterised by a constant concentration and can be interpreted as the area above the actual crystallisation process. Here the particles are freely floating with little or no interaction. Area B is the first indicator of a crystallisation process and shows an increased concentration just above an extended depletion zone (C). B and C are the areas that “feed” the increasing concentration region (D), often referred to as fan, that then eventually leads to the crystalline area E.

Furthermore a direct measure for the relative change in concentration can be extracted by evaluating the location of the minimum of the real-space correlation function at each height in the sample (dashed white line). The *ξ*-value of the minimum within a real-space correlation function depends, above a sufficient concentration to detect next neighbour correlations, on the concentration of microspheres within the probed region. An increase in concentration causes a shift of the minimum towards smaller *ξ*-values, because the next neighbour correlation implies a signal increase at even shorter distances, i.e. correlation lengths. Using this approach we can determine a relative concentration profile like indicated by the dashed white line in Fig. 6b.

## Discussion

The concentration profile presented in Fig. 6b can be compared to the confocal microscopy study presented by Sandorminski *et al*.^18^. The system used in ref. 18 is highly equivalent even though the colloids had to be modified with fluorescent molecules to enable fluorescent microscopy, which is not the case for the here presented study with *ξDFI*. Furthermore the highly penetrable nature of neutrons allows us to investigate a system larger than the ones accessible to light microscopy with the additional benefit of non-ionisig radiation that keeps the system chemically stabel during exposure. The shape of the dashed white line in Fig. 6b, indicating the position of the minimum of the real-space correlation function as a function of height in the sample, can directly be compared with Fig. 3 in ref. 18 and good agreement is found.

The combined nature of imaging and scattering data in *ξDFI* makes it possible for us to also directly compare the results to scattering studies without spatial resolution. The SESANS technique, which uses the interference of neutron spin states as contrast mechanism, is an ideal candidate for comparison, because it also offers real-space correlation functions as a result. Krouglov *et al*.^32^ have studied a similar system to the one presented here and identified, in three separate samples of different particle concentrations, three phases of colloidal systems: gas-like, liquid-like and a densely packed crystalline phase. With the additional spatial resolution in *ξDFI* we could reproduce these results in a single sample and could hence further identify the individual location of those phases in the heterogeneous system and thereby study the transition between them in detail.

The evolution of particle concentration as well as the phase transition from a disordered to an ordered state is also consistent with observations made in ref. 35 where the concentration of spheres in solution is determined as a crucial factor for the formation of colloidal crystals of nanoparticles. While the optical method does not allow for the study of microparticles, such as the ones used in our study, we do see that the initial concentration of 11.9% of microspheres in solution supports the formation of a crystalline phase. The combination of low Peclet number, together with some electrostatic screening, that leads to a softer interaction than for hard spheres, enables the particles to find their energetically most preferential packing and end up in a crystalline structure. These factors have been identified in ref. 36 to be crucial for crystallisation. Our study confirms such behaviour also for microspheres.

The agreement of the presented results with previous studies from both imaging and scattering experiments illustrate the combined information content in *ξDFI*. Previously reported approaches of quantitative DFI^6^ which utilize wavelength dispersive measurements to probe a certain range of correlation lengths *ξ* would not qualify for the current study, because the increase of the scattering cross-section with the wavelength would lead to a signal saturation due to dynamic range limitations at high concentrations and long correlation lengths. With the novel experimental setup, utilizing the third Talbot order, we could also extend the accessible *ξ* range from previously approx. 1.5 μm–2.5 μm^12^ to 0.9 μm–4.3 μm. Only this approach enabled the study of the crystallisation processes by accessing the structure factor impact on the real space scattering function.

Our study underlines the potential of combining scattering and imaging information for the investigation and the understanding of crystallisation processes. Most importantly the spatially resolved nature of retrieved information is particularly well suited for the investigation of heterogeneous crystallisation. The method hence has great potential for future studies of a variety of systems to understand crystallisation processes that are not necessarily driven by gravity but happen e.g. at initial seeds. *ξDFI* and the experimental and evaluation procedure presented here illustrates the potential for applications in numerous fields where microstructural changes appear on a macroscopical scale. The applications range from the behaviour of biological systems, such as bacteria, to cutting edge technology with the production of photonic crystals.

## Methods

### Neutron grating interferometry in the third Talbot distance

The experiments presented here were conducted at the cold neutron imaging beamline ICON at the Paul Scherrer Institut (PSI) in Switzerland. Image acquisition was done with a traditional imaging setup with a sCMOS camera (Andor IKON Neo) and a 200 6LiF/ ZnS scintillator. A turbine based energy selector was used to get a monochromatic beam at a wavelength of 4.1 Å with a wavelength resolution of 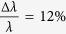. Its impact on the derived autocorrelation length is discussed in Supplement 3. We used an exposure time of 50 s for each image of the 17 steps within the nGI scan and thus utilized the dynamic range of the camera optimally.

For this study we designed a new neutron grating interferometer that images in the third Talbot distance. The grating periods were set to be 365 μm for G0, 7.91 μm for G1 and 4 μm for G2. Those settings lead to a Talbot distance of ~5.7 cm and a G0–G1 distance of 5.23 m. The source grating (G0) consists of a 20 μm layer of gadolinium, that was sputtered on a quartz wafer. The grating structure was lasered in the Gd layer. G1 acts as a phase grating and was thus produced out of a Si wafer using the Bosch process. The final absorption grating (G2) was manufactured as presented in ref. 15.

The relatively large distance of ~5.7 cm between G1 and G2 allowed us to place and scan the sample between them. This way we could move the sample between 0.9 and 4.3 cm distance from G2. The limits were set by the sample holder.

### Face-centered model for colloidal spheres

As suggested by studies such as refs 32 and 36 one likely crystalline phase for gravity driven sedimented colloids is the face-centered cubic structure. The calculated function presented here assumes a particle diameter of 3 μm, with an effective nearest neighbour separation of 4.5 μm to compensate for the electrical double layer formed by charges on the colloidal surface and a distortion factor of 0.1 to include the effect of brownian motion.

## Additional Information

**How to cite this article**: Harti, R. P. *et al*. Sub-pixel correlation length neutron imaging: Spatially resolved scattering information of microstructures on a macroscopic scale. *Sci. Rep.* **7**, 44588; doi: 10.1038/srep44588 (2017).

**Publisher's note:** Springer Nature remains neutral with regard to jurisdictional claims in published maps and institutional affiliations.

## Supplementary Material

Supplementary Information
