# Supplementary material for: Sub-pixel correlation length neutron imaging: Spatially resolved scattering information of microstructures on a macroscopic scale
Source: Sci Rep. 2017 Mar 17;7:44588. doi: 10.1038/srep44588 (PMC5355987; doi:10.1038/srep44588)
Supplement: Supplementary Information [file srep44588-s1.pdf]

Sub-pixel correlation length neutron imaging: Spatially resolved scattering information of microstructures on a macroscopic scale

Ralph P. Harti[1,2*], Markus Strobl[3,4], Benedikt Betz[1],
Konstantins Jefimovs[5,6], Matias Kagias[5,6], Christian Gruenzweig[1]

[1]Laboratory for Neutron Scattering and Imaging, Paul Scherrer Institut, 5232 Villigen, Switzerland
[2]University of Geneva, 1211 Geneva, Switzerland
[3]European Spallation Source E.R.I.C., 22100 Lund, Sweden
[4]Niels Bohr Institute, Copenhagen University, 2100 Copenhagen, Denmark
[5]Swiss Light Source, Paul Scherrer Institut, 5232 Villigen, Switzerland
[6]Institute for Biomedical Engineering, ETH Zurich, 8092 Zurich, Switzerland

[*]To whom correspondence should be addressed; E-mail: Ralph.Harti@psi.ch.

December 19, 2016

Supplementary Material

Supplement 1

In order to verify the diameter of the particles used we followed the approach of preparing a sample of isolated particles in a density matched solution of 59 % water and 41 % heavy water. This leads to a system that does not settle and contains 8.55 wt. % of microspheres. Figure S 1 shows both the experimental data and the model that described 3 micrometer isolated spheres.

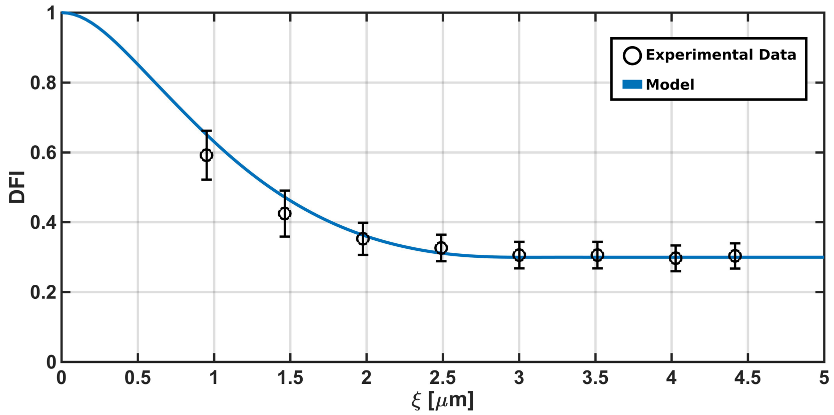

Figure S 1: The change in DFI value as a function of the autocorrelation length ξ. The black data points are fitted with a model for 3 micrometer isolated spheres.

The model used for describing the experimental data is the same as described in Equation 3 in the manuscript. No structure factor was included and a purely isolated system of hard spheres is considered. The data is in good agreement with the behaviour expected for the particles that were purchased as NIST traceable standard with well defined diameter.

Supplement 2

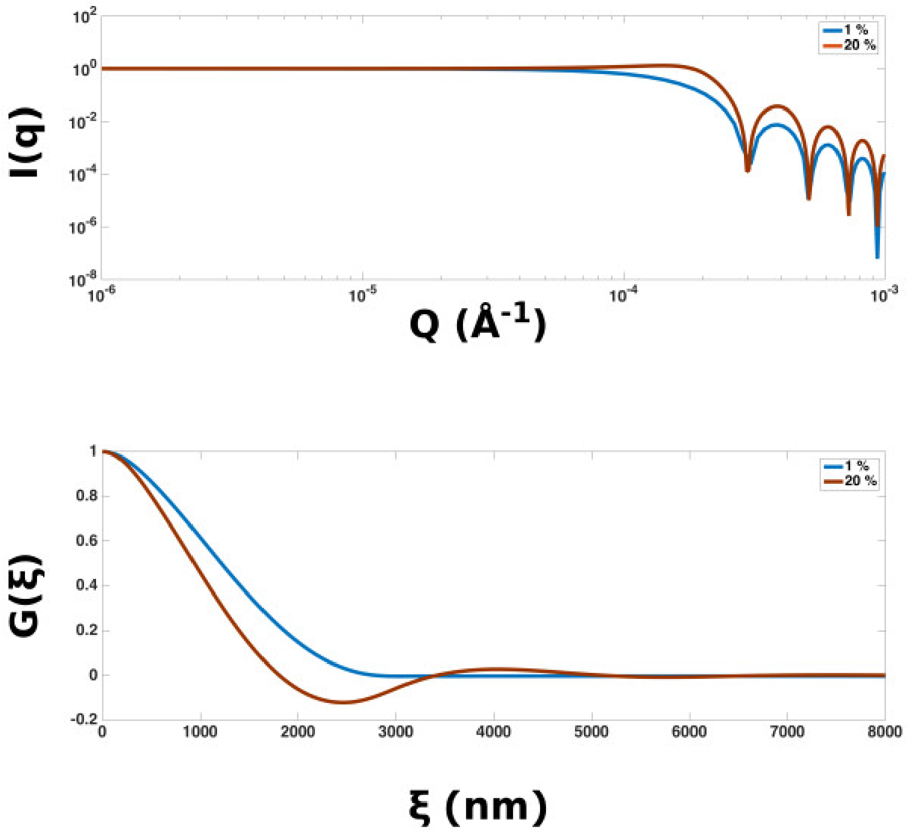

Figure S 2: Comparison of change in power spectrum and autocorrelation function as a consequence of increased colloidal concentration. The autocorrelation function is determined via a hankel transform of the power spectrum and uses the Perkus-Yevick approximation to model inter-particle correlations. Models for various power spectra are available in the small angle scattering software SASView.

Supplement 3

The energy distribution after the velocity selector is best described as a gaussian function (see Figure S 3) of the form:

$$f(x) = ae^{-((x-b)^2)/2c^2)}$$

With the fitting parameters $a = 1$, $b = 4 \cdot 10^{-10}$ and $c = 0.49 \cdot 10^{-10}$. This leads to $\frac{\Delta\lambda}{\lambda} = 12\%$.

As the autocorrelation length is described by a linear relation to the wavelength ($\xi = \frac{\lambda L}{p2}$) the resulting shape of autocorrelation length distributions also resembles a gaussian. Consequently the measured autocorrelation length (ξ) during the measurement is also best described by

$$\frac{\Delta\xi}{\xi} = 12\%$$

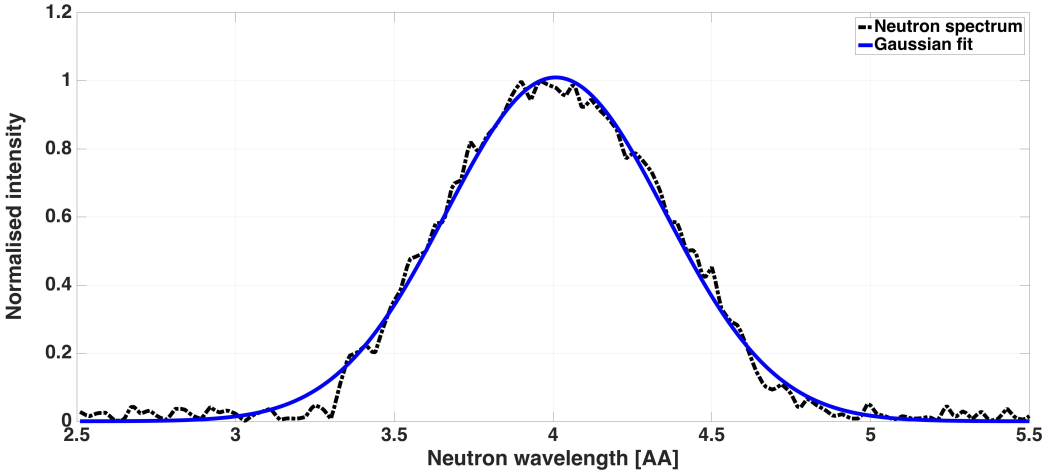

Figure S 3: Measured neutron spectrum at the ICON beamline using a velocity selector set to 4 Å. The neutron spectrum is fitted with a gaussian distribution and has been normalised to the maximum intensity.
